# Gain and Loss Learning Differentially Contribute to Life Financial Outcomes

**DOI:** 10.1371/journal.pone.0024390

**Published:** 2011-09-06

**Authors:** Brian Knutson, Gregory R. Samanez-Larkin, Camelia M. Kuhnen

**Affiliations:** 1 Department of Psychology, Stanford University, Stanford, California, United States of America; 2 Psychological Sciences, Vanderbilt University, Nashville, Tennessee, United States of America; 3 Finance Department, Kellogg School of Management, Evanston, Illinois, United States of America; University of Minnesota, United States of America

## Abstract

Emerging findings imply that distinct neurobehavioral systems process gains and losses. This study investigated whether individual differences in gain learning and loss learning might contribute to different life financial outcomes (i.e., assets versus debt). In a community sample of healthy adults (n = 75), rapid learners had smaller debt-to-asset ratios overall. More specific analyses, however, revealed that those who learned rapidly about gains had more assets, while those who learned rapidly about losses had less debt. These distinct associations remained strong even after controlling for potential cognitive (e.g., intelligence, memory, and risk preferences) and socioeconomic (e.g., age, sex, ethnicity, income, education) confounds. Self-reported measures of assets and debt were additionally validated with credit report data in a subset of subjects. These findings support the notion that different gain and loss learning systems may exert a cumulative influence on distinct life financial outcomes.

## Introduction

What promotes wealth? Specifically, why do some people accrue assets while others accumulate debt? While environmental factors (e.g., family socioeconomic status and inheritance) undoubtedly play powerful roles in determining life financial outcomes [1], some of those born into poverty eventually amass riches, while others who are born into wealth squander their inheritance. Do life financial outcomes depend solely upon chance, or can individual differences exert a subtle yet persistent influence over time?

Individuals reliably vary in both their cognitive and affective capacities [2]. While some evidence suggests that individual differences in cognitive capacities may influence financial preferences and outcomes [3], [4], [5], less research has focused on individual differences in affective capacities. Even if individual differences in affect can influence life financial outcomes, it is not clear whether such an influence might arise from single or multiple systems (e.g., one which anticipates gain and another which anticipates loss).

Neurobehavioral methods can help investigators to distinguish whether single or multiple systems drive seemingly unitary behaviors. For instance, a growing body of neuroeconomic findings suggests that two distinct neural systems associated with affect (rather than one) can influence subsequent choice. On the one hand, neural activity associated with positive aroused feelings (e.g., “excitement”) in the nucleus accumbens (NAcc) and medial prefrontal cortex (MPFC) precedes acceptance of risky gambles and purchase of products [6], [7], and can facilitate learning about gains [8], [9], [10]. On the other hand, neural activity associated with negative aroused feelings (e.g., “anxiety”) in the anterior insula precedes rejection of risky gambles and refusal to purchase products [6], [7], [11], and also facilitates learning about losses [12], [13], [14]. Individual differences in recruitment of these circuits has also been linked to individual differences in learning [15], [16], [17], [18].

Beyond their momentary influence, individual differences in learning about gain and loss might eventually alter life financial outcomes. While traditional finance considers the balance of assets and debts (i.e., the debt to asset ratio) as a measure of personal wealth, a multiple systems view implies that people may instead frame and maintain separate “mental accounts” [19] associated with gains and losses [20]. Individual differences in learning about gains might then preferentially enhance peoples' ability to recognize and acquire potential gains (which accrue in the form of assets), while individual differences in learning about losses might instead enhance peoples' ability to detect and avoid losses (which minimizes debt). Importantly, such an account assumes only that gain and loss learning can independently vary, which could then allow gain learning to correlate with high assets, but loss learning to distinctly correlate with low debt.

In the present study, we tested whether single or dual learning systems might contribute to life financial outcomes. Specifically, we examined whether individual differences in gain learning and loss learning were distinctly associated with assets and debt, respectively. To do so, we controlled for potential cognitive and socioeconomic confounds, and also validated self-reported measures of assets and debt with credit report data in half of the sample.

## Materials and Methods

### Subjects

A survey research firm initially contacted individuals who were representative of San Francisco peninsula residents with respect to sex, income, education, ethnicity, and occupation. Because a different aim of the study focused on aging, subjects were evenly sampled across the life span and screened for dementia (i.e., with Mini Mental scores >26). Seventy-five healthy adults (age range = 20–85) participated (see summary statistics of individual difference variables in Table 1). Written informed consent was obtained from all subjects, and the study was approved by the Institutional Review Board of the Stanford University School of Medicine. An additional seven subjects (not included in the 75 subjects listed above) initially participated, but did not report full socioeconomic, risk preference, and financial data and were excluded from all analyses. Subjects received fixed payment of $20 per hour, as well as cash equivalent to their total earnings in the task. Subjects were also informed that they could lose money on the task, and that any losses they accrued would be deducted from their total earnings. Subjects completed the self-report measures before completing the learning task. To validate self-report measures of assets and debt, credit reports were obtained for approximately half of the sample who agreed to provide their report and for whom credit reports could be obtained after the experiment (n = 37).

**Table 1:** Summary of individual difference variables.

Variable	Mean (SD)
Age (years)	54.25 (16.67)
Education (years)	15.44 (2.84)
Sex	41 male/34 female
Working Memory (score)	14.04 (3.03)
Cognitive Flexibility (secs)	34.21 (15.51)
Numeracy (score)	7.92 (1.37)
Overall correct choices (%)	0.61 (0.21)
Gain correct choices (%)	0.58 (0.36)
Loss correct choices (%)	0.63 (0.21)
Risk Aversion (indiff. pt.)	5.45 (3.19)
Loss Aversion (indiff. pt.)	7.03 (4.26)
Income	6.73 (2.37)
Debt	7.48 (5.12)
Assets	12.96 (3.85)

The working memory score indexes the number of items that subjects can hold in memory, the cognitive flexibility score represents the additional time required to connect alternating numbers and letters versus sequential numbers, and the numeracy score represents the number of correct answers out of 11 total items. Risk aversion and loss aversion are indices between 0 and 12 that represent the switching point in lottery questions involving choices between sure outcomes and gambles (see Supplementary Methods). Income, debt, and assets are based on ordered categories (e.g., an income rating of 6 corresponds to an average household income of $60,000–$79,000 and a rating of 7 corresponds to $80,000–$99,000; a debt rating of 7 corresponds to $20,000–$39,999 and a rating of 8 corresponds to $40,000–$59,999; and an assets rating of 13 corresponds to $200,000–$499,999).

### Monetary Incentive Learning (MIL) Task

**Figure 1:**
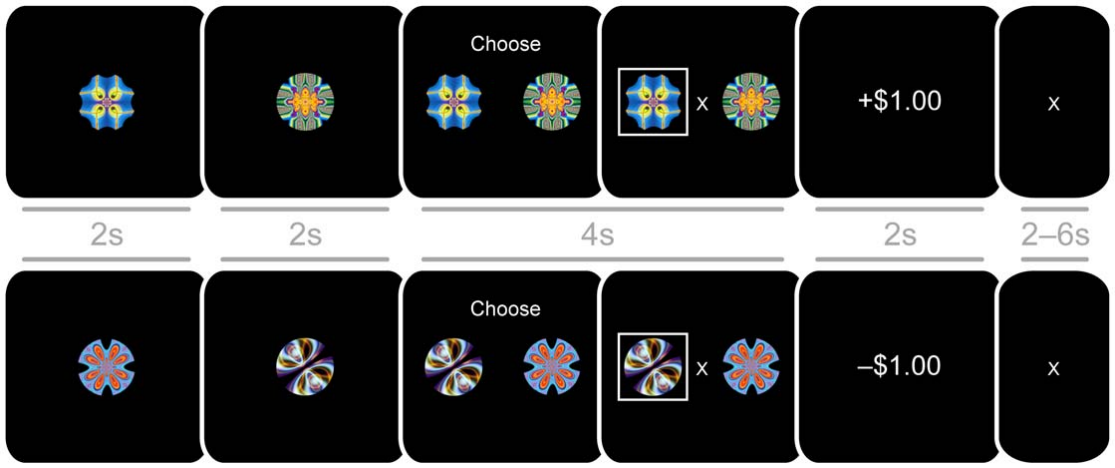
Trial structure for Monetary Incentive Learning task gain (top) and loss (bottom) conditions.

Behavioral measures of gain learning and loss learning were elicited with a probabilistic learning task designed to explicitly separate gain and loss conditions (Figure 1). The MIL task was adapted from conventional reinforcement learning tasks [10], [21], [22]. Subjects saw and chose between one of three pairs of fractal cues (gain acquisition, loss avoidance, or neutral) in each run of 12 trials per condition, for a total of 36 trials. After choosing one of the cues from a pair, subjects saw the outcome associated with their choice. On average, one of the cues yielded a better outcome, while the other yielded a worse outcome. In gain cue pairs, the better cue had a higher probability of returning gains (66% +$1.00 and 33% +$0.00) than the worse cue (33% +1.00 and 66% +$0.00); while in loss cue pairs, the better cue had a higher probability of returning nonlosses (66% –$0.00 and 33% –$1.00) than the worse cue (33% –$0.00 and 66% –$1.00). In neutral cue pairs, choice of either cue had no impact on outcomes (100% $0.00). Thus, the only difference between the gain and loss learning conditions involved the valence of the information presented (i.e., gain versus loss). Since the probabilistic learning component was identical across task conditions, differential performance could be attributed to affective gain versus loss framing of different conditions.

Within each cue pair, cues appeared randomly and with equal frequency on the left or right side of the screen. The computer randomly assigned each cue to either the better or worse outcome distribution at the beginning of each run in a counterbalanced fashion. Different cue pairs were used for practice and experimental sessions in order to minimize memory-related interference. Subjects were explicitly informed about cue probabilities before the practice session and told to try to maximize their earnings throughout the experiment. Subjects received cash for their performance after the experimental sessions, but not the practice sessions.

Measures of gain learning and loss learning performance were assessed by calculating the percentage of choices that matched the “correct” cue (i.e., or had the higher probability of an advantageous outcome) in each condition (see Supplementary Figure S1. Subjects' percentage “correct” choices in the gain and loss conditions (excluding the first trial) were used as primary predictors of life financial outcomes. Based on information that each subject received during the task (i.e., observed outcomes), a measure of “optimal” choice can be computed as the fraction of trials where a subject made the correct ex-ante Bayesian choice (excluding trials in which either option had an equal chance of being optimal, such as the first trial; see Supplementary Methods S1). This “optimal” choice metric was computed for each individual in each condition and used to validate the simpler “correct” choice measure. Supporting the validity of the simpler “correct” choice measures, gain optimal choices (but not loss optimal choices) were associated with gain correct choices, while loss optimal choices (but not gain optimal choices) were associated with loss correct choices (see Supplementary Table S1). Supporting the distinctness of gain and loss learning, gain and loss correct choices were not significantly correlated within subject (r = 0.09, n.s.).

### Life Financial Outcomes

Assets and debt were assessed via self-report in all subjects (n = 75) and validated with credit report information on a subset of subjects (n = 37). Assets were assessed with the question: “What are your approximate current assets? (i.e., portion of home owned, bank accounts, investments, belongings)” using a 16-category ordinal response scale ranging from <+$500.00 in the lowest category to >+$1,500,000.00 in the highest. Debt was assessed with the question: “What are your approximate current debts? (i.e., outstanding home loans, outstanding car loans, outstanding student loans, credit card debt, medical debt)” using a 16 category ordinal response scale ranging from <$500.00 in the lowest category to >$1,500,000.00 in the highest.

From credit reports of the subsample, we extracted the overall credit score (also known as the FICO score), which is a proprietary index commonly used in the United States to determine creditworthiness [23]. As expected, the debt-to-asset ratio derived from self reported assets and debt was significantly associated with the credit score (r = −0.48, p<.01) such that subjects with lower debt-to-asset ratios had higher credit scores. We also specifically computed the available credit amount (i.e., the sum of the credit limits of all open accounts) and the percent of credit used (i.e., the sum of credit used divided by the sum of the credit limits of all open accounts) from the information contained in the credit reports. These measures were used to distinguish and validate self-reported assets and debt. Supporting the validity of the self-reported measures of assets and debt, assets were associated with available credit, whereas debt was associated with percent of credit used (see Supplementary Table S2).

### Cognitive and Socioeconomic Measures

Selected neuropsychological tests were administered to assess potential cognitive confounds. The WAIS-III Digit Span Test assessed working memory capacity by requiring subjects to repeat numerical strings forward and backwards. Working memory capacity is highly correlated with and often used to index general intelligence [24]. The Trail Making Test (TMT) assessed cognitive flexibility by requiring subjects initially to sequentially connect circled numbers, and then to connect a series of alternating numbers and letters [25]. Finally, a numeracy inventory (11 items) assessed quantitative skills with basic number problems [26]. Socioeconomic variables including age (years), education (8 ordinal category scale), sex (M/F), ethnicity (open-ended), and income (a 16 level ordinal scale with the same categories used for assets and debt) were also assessed via self-report.

### Risk Preference Measures

Two sets of questions (12 items each) assessed risk aversion and loss aversion by soliciting subjects' preferences between probabilistic or “risky” gambles and certain or “safe” amounts of money. For both risk aversion and loss aversion measures, a number was assigned (i.e., an integer lower or equal to 12, representing one of the items in descending order) which corresponded to the item on which each subject switched from preferring the safe to preferring the risky option (see Supplementary Methods S2). Neither risk aversion nor loss aversion measures correlated significantly with gain learning or loss learning measures.

### Analyses

Analyses included multiple regression models constructed to test predicted relationships between learning variables and life financial outcomes. Reduced regressions first tested the association between learning variables (i.e., the average of gain and loss % correct choices, gain % correct choices, loss % correct choices) and life financial outcomes (i.e., debt to asset ratio, assets, debt). Full regressions then verified the robustness of these same relationships after controlling for potential socioeconomic (i.e., income, age, education, sex, ethnicity), cognitive (working memory, cognitive flexibility, numeracy), and risk preference (i.e., risk aversion, loss aversion) confounds.

## Results

An initial set of regression models tested whether general learning (i.e., which combined performance across gain and loss learning conditions) could account for accumulated debt-to-asset ratio, as well as assets and debt separately (Table 2). The simple model relating overall correct choices to debt-to-asset ratio was significant (R^2^  = .05, p<.05) and revealed a negative association of overall correct choices with debt-to-asset ratio (β = −0.92, t = −2.20, p<.05). The corresponding full model (including socioeconomic, cognitive, and risk preference variables) was also significant (R^2^ = .18, p<.001), but the negative association of overall correct choices with debt-to-asset ratio was reduced to marginal significance (β = −0.82, t = −1.85, p<.10). Further, overall correct choices were not significantly associated with assets or debt separately.

**Table 2:** Relationships of general learning with debt-to-asset ratio, assets, and debt.

	Debt-to-Asset Ratio	Debt-to-Asset Ratio (full model)	Assets	Assets (full model)	Debt	Debt (full model)
Overall correct choices	**−0.92 (0.42)** –2.20*	**−0.82 (0.44) –1.86**	3.92 (2.03) 1.92	2.52 (1.48) 1.70	−0.69 (2.84) –0.24	−2.11 (3.14) –0.67
Debt	—	—	0.13 (0.09) 1.50	0.08 (0.06) 1.37	—	—
Assets	—	—	—	—	0.24 (0.16) 1.50	0.36 (0.27) 1.37
Income		−0.07 (0.04) –1.65		0.63 (0.15) 4.27***		0.35 (0.35) 0.99
Working memory		−0.03 (0.04) –0.68		0.04 (0.13) 0.34		−0.23 (0.26) –0.88
Cognitive flexibility		0.00 (0.01) 0.19		0.02 (0.02) 0.97		0.01 (0.05) 0.15
Numeracy		0.05 (0.07) 0.67		0.30 (0.24) 1.24		0.28 (0.51) 0.55
Risk aversion		0.03 (0.03) 0.84		0.11 (0.10) 1.08		0.02 (0.22) 0.08
Loss aversion		0.01 (0.02) 0.65		−0.12 (0.07) –1.64		−0.05 (0.16) –0.31
Age		−0.02 (0.01) –2.26*		0.16 (0.02) 6.72***		−0.07 (0.06) –1.05
Education		0.04 (0.04) 0.94		−0.02 (0.14) –0.17		0.30 (0.29) 1.02
Sex = male		−0.09 (0.19) –0.49		−0.22 (0.64) –0.34		1.26 (1.31) 0.96
Ethnicity = Af-Am		0.38 (0.56) 0.67		0.82 (1.95) 0.42		6.26 (3.96) 1.58
Ethnicity = Hisp		0.72 (0.29) 2.50*		−1.86 (0.99) –1.88		3.84 (2.05) 1.87
Ethnicity = As-Am		0.19 (0.26) 0.75		−0.02 (0.87) –0.03		1.44 (1.79) 0.81
Ethnicity = Other		−0.26 (0.56) –0.47		0.31 (1.91) 0.17		3.10 (3.93) 0.79
Constant	1.26 (0.27) 4.69***	1.53 (1.01) 1.51	9.62 (1.45) 6.63***	−4.84 (3.41) –1.42	4.82 (2.44) 1.97	0.01 (7.19) 0.00
R^2^	.06*	.34*	.08	.69***	.03	.24
Adjusted R^2^	.05*	.18*	.05	.61***	.00	.05
Observations	75	75	75	75	75	75

Values listed are coefficient (s.e.m.) t-statistic. * p<.05, ** p<.01, ***p<.001; predicted associations in **bold**.

A second set of regression models tested the key predictions that gain learning would specifically correlate with assets but loss learning would specifically correlate with debt (Table 3). Thus, both gain and loss correct choices were included as independent variables in these regression models. Because of their moderate positive correlation (r = .21, p<.05; suggesting partial independence), assets were included in models that accounted for debt and vice-versa. Consistent with the notion that gain learning and loss learning promote more specific life financial outcomes, neither the simple nor the full regression models relating gain correct choices and loss correct choices to debt-to-asset ratio were significant.

**Table 3:** Relationships of gain and loss learning with debt-to-asset ratio, assets, and debt.

	Debt-to-Asset Ratio	Debt-to-Asset Ratio (full model)	Assets	Assets (full model)	Debt	Debt (full model)
Gain correct choices	−0.42 (0.25) –1.67	−0.27 (0.26) –1.03	3.95 (1.18) 3.34**	2.39 (0.86) 2.77**	2.46 (1.72) 1.43	1.94 (1.85) 1.05
Loss correct choices	−0.57 (0.44) –1.30	−0.81 (0.46) –1.74	−3.56 (2.12) –1.68	−2.15 (1.62) –1.33	−7.01 (2.85) –2.46*	−8.62 (3.15) –2.74**
Debt	—	—	0.04 (0.09) 0.49	0.03 (0.06) 0.44	—	—
Assets	—	—	—	—	0.08 (0.16) 0.49	0.12 (0.27) 0.44
Income		−0.07 (0.04) –1.64		0.67 (0.14) 4.67**		0.52 (0.34) 1.54
Working memory		−0.02 (0.04) –0.55		0.07 (0.12) 0.56		−0.14 (0.25) –0.55
Cognitive flexibility		0.00 (0.01) 0.05		0.02 (0.02) 0.70		−0.00 (0.05) –0.11
Numeracy		0.05 (0.07) 0.72		0.35 (0.23) 1.51		0.43 (0.48) 0.89
Risk aversion		0.02 (0.03) 0.66		0.07 (0.10) 0.72		−0.05 (0.21) –0.25
Loss aversion		0.01 (0.02) 0.59		−0.14 (0.07) –1.91		−0.10 (0.15) –0.69
Age		−0.02 (0.01) –2.43*		0.14 (0.02) 6.09***		−0.06 (0.06) –1.02
Education		0.04 (0.04) 0.92		−0.01 (0.14) –0.10		0.28 (0.28) 1.03
Sex = male		−0.09 (0.19) –0.49		−0.14 (0.62) –0.22		1.26 (1.25) 1.01
Ethnicity = Af-Am		0.30 (0.57) 0.52		0.54 (1.88) 0.29		5.05 (3.78) 1.34
Ethnicity = Hisp		0.73 (0.29) 2.54*		−1.57 (0.96) –1.64		3.69 (1.95) 1.90
Ethnicity = As-Am		0.15 (0.26) 0.58		−0.28 (0.85) –0.33		0.68 (1.73) 0.40
Ethnicity = Other		−0.32 (0.56) –0.56		0.06 (1.84) 0.03		2.32 (3.75) 0.60
Constant	1.31 (0.32) 4.15***	1.79 (1.04) 1.71	12.59 (1.71) 7.35***	−2.75 (3.40) –0.81	9.43 (2.92) 3.23**	3.88 (6.97) 0.56
R^2^	.06	.35*	.18**	.71***	.12*	.33**
Adjusted R^2^	.04	.18*	.14**	.64***	.08*	.14**
Observations	75	75	75	75	75	75

Values listed are coefficient (s.e.m.) t-statistic. * p<.05, ** p<.01, ***p<.001; predicted associations in **bold**.

As predicted, however, the simple regression model relating gain learning to assets was significant (R^2^ = .14, p<.01), revealing a positive association of gain correct choices (but not loss correct choices) with assets (β = 3.95, t = 3.34, p<.01). The corresponding full model (including socioeconomic, cognitive, and risk preference variables) was also significant (R^2^ = .64, p<.001), and continued to demonstrate a positive association of gain correct choices with assets (β = 2.39, t = 2.77, p<.05). Of the control variables, only age and income were also significantly positively associated with assets (ps<.01).

The simple regression model relating loss learning to debt was also significant (R^2^ = .08, p<.05), revealing a negative association of loss correct choices (but not gain correct choices) with debt (β = −7.01, t = −2.46, p<.05). The corresponding full model (including socioeconomic, cognitive, and risk preference variables) continued to demonstrate a negative association of loss correct choices with debt (β = −8.62, t = −2.74, p<.01). None of the control variables in this model were significantly associated with debt.

When Bayesian optimal learning measures were substituted for simpler gain percent correct and loss percent correct measures, similar results were obtained. Specifically, in the full model, gain optimal learning was associated with assets (β = 2.27, t = 2.35, p = .02), but not debt (β = 2.76, t = 1.42, p = .16), while loss optimal learning was associated with debt (β = −9.38, t = −3.01, p = .004), but not assets (β = −1.08, t = −0.63, p = 0.53).

## Discussion

**Figure 2:**
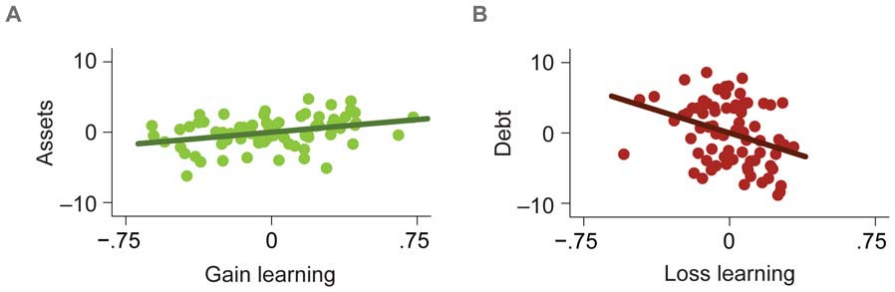
Individual differences in gain learning account for assets (A) and in loss learning account for debt (B). Panels A–B depict plots in which trendlines indicate the correlation between residuals (y-axis values represent rescaled residuals after controlling for the covariates listed in Table 3).

These findings provide initial evidence connecting incentive learning to long-term financial outcomes. They validate an experimental learning task by indicating that it can elicit behaviors related to real-world financial choice. Beyond linking general learning to financial well-being, the findings support a more specific account in which gain learning promotes asset accumulation, while loss learning promotes debt avoidance. Remarkably, individual differences in socioeconomic, cognitive, and risk preference variables could not account for these associations. The findings are thus consistent with an account in which distinct gain and loss learning systems influence not only immediate choice but also long-term financial outcomes (Figure 2).

Although the observed associations are predicted, specific, and robust, they are not causal. According to a “third variable” alternative interpretation, other individual difference variables related to socioeconomic status, cognitive capacities, and risk preferences may play more prominent roles in determining life financial outcomes. For instance, because age has been related to probabilistic learning, it might also account for associations between learning performance and life financial outcomes [27], [28]. In the present study, however, confounds related to socioeconomic status, cognitive capacity (including age), and risk preferences could not account for the predicted links between gain learning and asset accumulation or between loss learning and debt avoidance (Table 3).

An alternative “reverse causality” account might posit that greater assets increase gain learning, while higher debt increases loss learning. Based on the economic notion of diminishing marginal returns, however, it seems unlikely that increased assets would enhance (rather than blunt) individuals' sensitivity to gains [29]. Additionally, in an independent sample (n = 30), gain learning and loss learning performance in the MIL task showed two week test-retest reliability that did not change significantly over repeated administrations (r = .50 for gain correct choices and r = .49 for loss correct choices), consistent with moderate stability over time. Only future longitudinal investigations will be able to causally determine whether gain learning and loss learning influence subsequent life financial outcomes.

The primary outcomes in this study included general self-reported measures of accumulated assets (including the savings accounts, home value, etc.) and debt (including credit card balances, mortgage balance, etc.). Future studies might profitably explore the relationship between gain and loss learning performance and more specific categories of assets and debt. For instance, individual differences in gain learning might be more strongly associated with the value of risky investments than with home value, whereas individual differences in loss learning might be more strongly associated with credit card debt than with outstanding mortgage debt. Indirect evidence does suggest that the association between loss learning and overall debt is not determined by the mortgage component of debt, since adding subjects' current home value (a likely correlate of mortgage debt) to regression models in an auxiliary analysis did not change either the significant association between loss learning and low debt (p<.01), or the lack of association between gain learning and debt (i.e., for the 82% of subjects who were homeowners).

This research uniquely spans multiple levels of analysis and timescales, linking behavior in the laboratory to significant long-term financial outcomes, and so can offer a number of advances over previous work. Specifically, we recruited a community sample with significant assets and debt rather than a sample of convenience, validated measures of life financial outcomes with credit report data on a subsample, assessed and controlled for other potentially important individual difference confounds, and were able to demonstrate selective dissociations between gain and loss learning.

In conclusion, these findings demonstrate an association between learning and life financial outcomes, and suggest that individual differences in gain and loss learning may systematically alter assets and debt respectively – even beyond external social and economic forces. Specifically, sensitivity to gain information may promote approach towards financial opportunities, while sensitivity to loss information may instead promote avoidance of financial threats. By extension, these learning mechanisms may also move people not only towards different choices but also towards different financial choice environments that advertise the presence of opportunities (e.g., casinos) or the absence of threats (e.g., insurance agencies). The elucidation of individual differences in distinct gain and loss learning mechanisms implies that imbalances in affective learning could eventually create chronic biases in choice. Fortunately, assessment of these biases may resolve targets for intervention – either on the part of individuals or their financial advisors.

## Supporting Information

Figure S1 **Gain and loss learning over time for fast learners versus slow learners.** Subjects were median split by overall gain learning (high vs. low performance) and median split by overall loss learning (high vs. low performance). The vertical axis represents the proportion of subjects who chose the high probability cue on each trial (± S.E.M.).
(PDF)

Table S1 **Validation of gain and loss correct choices with optimal choice measures.** (correlation coefficients; *p<.05, **p<.01, ***p<.001, two-tailed, N = 75; related to Table 2).
(PDF)

Table S2 **Self-declared assets and debt correlate with distinct credit report variables.** (top entry: coefficient (S.E.M.); bottom entry: t-statistic; * p<.05, ** p<.01, two-tailed; related to Figure 2).
(PDF)

Methods S1 **Calculation of optimal learning measures based on Bayes**' **rule.**
(PDF)

Methods S2 **Calculation of risk aversion and loss aversion measures.**
(PDF)
