# Supplementary material for: Gain and Loss Learning Differentially Contribute to Life Financial Outcomes
Source: PLoS One. 2011 Sep 6;6(9):e24390. doi: 10.1371/journal.pone.0024390 (PMC3167846; doi:10.1371/journal.pone.0024390)

Figure S1. Gain and loss learning over time for fast versus slow learners. Subjects were median split by overall gain learning (high vs. low performance) and median split by overall loss learning (high vs. low performance). The vertical axis represents the proportion of subjects who chose the high probability cue on each trial (± S.E.M.).

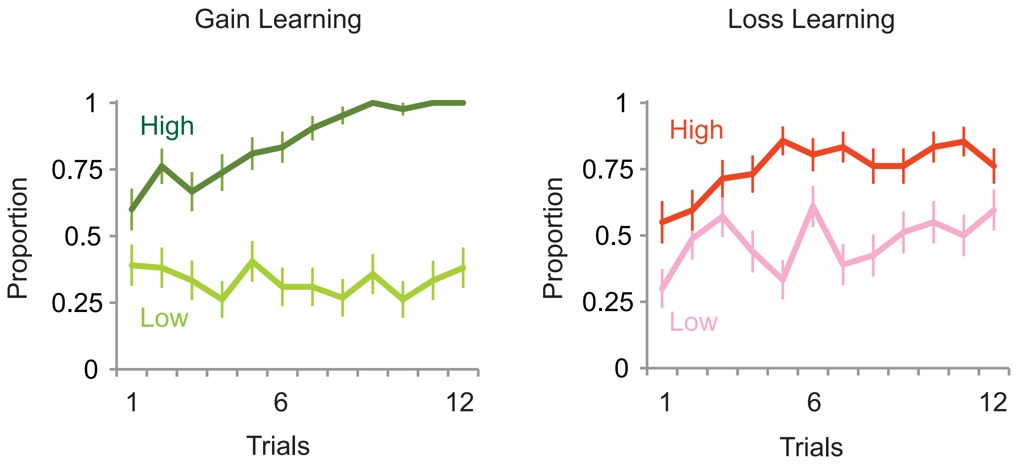

Supplement: Figure S1 — Gain and loss learning over time for fast learners versus slow learners. Subjects were median split by overall gain learning (high vs. low performance) and median split by overall loss learning (high vs. low performance). The vertical axis represents the proportion of subjects who chose the high probability cue on each trial (± S.E.M.). (PDF) [file pone.0024390.s001.pdf]
