# Supplementary material for: Gain and Loss Learning Differentially Contribute to Life Financial Outcomes
Source: PLoS One. 2011 Sep 6;6(9):e24390. doi: 10.1371/journal.pone.0024390 (PMC3167846; doi:10.1371/journal.pone.0024390)
Supplement: Table S1 — Validation of gain and loss correct choices with optimal choice measures. (correlation coefficients; *p<.05, **p<.01, ***p<.001, two-tailed, N = 75; related to Table 2). (PDF) [file pone.0024390.s002.pdf]

Table S1. Validation of gain and loss correct choices with optimal choice (correlation coefficients; *p<.05, **p<.01, ***p<.001, two-tailed, N = 75; related to Table 2).

	Gain percent choices	*Loss percent choices*
Gain optimal choices	0.92***	0.08
Loss optimal choices	0.03	0.71***
