# Supplementary material for: Gain and Loss Learning Differentially Contribute to Life Financial Outcomes
Source: PLoS One. 2011 Sep 6;6(9):e24390. doi: 10.1371/journal.pone.0024390 (PMC3167846; doi:10.1371/journal.pone.0024390)
Supplement: Table S2 — Self-declared assets and debt correlate with distinct credit report variables. (top entry: coefficient (S.E.M.); bottom entry: t-statistic; * p<.05, ** p<.01, two-tailed; related to Figure 2). (PDF) [file pone.0024390.s003.pdf]

Table S2. Self-declared assets and debt correlate with distinct credit report variables. (top entry: coefficient (S.E.M.); bottom entry: t-statistic; * p<.05, ** p<.01, two-tailed; related to Figure 2)

	Assets	*Debts*
Credit amount	4.79×10^{-6} (0.00)<br>2.37*	6.64×10^{-6} (0.00)<br>3.39**
Percent credit used	–2.12 (1.34)<br>–1.58	7.18 (1.29)<br>5.56***
Constant	13.62 (0.78)<br>17.39***	2.40 (0.76)<br>3.17**
Adj. R^2	.11	.63
Observations	37	37
