# Supplementary material for: Gain and Loss Learning Differentially Contribute to Life Financial Outcomes
Source: PLoS One. 2011 Sep 6;6(9):e24390. doi: 10.1371/journal.pone.0024390 (PMC3167846; doi:10.1371/journal.pone.0024390)
Supplement: Methods S1 — Calculation of optimal learning measures based on Bayes' rule. (PDF) [file pone.0024390.s004.pdf]

Methods S1. Calculation of optimal learning measures based on Bayes' rule

For each subject, we calculated the fraction of trials where they chose in accordance with Bayes' rule (i.e., they picked the asset which had the higher Bayesian posterior probability of being the good one, conditional on the information known by the subject prior to choice). The calculation of the Bayesian posterior probability that a particular asset (or cue) was the good one was implemented as follows: Assume one of assets A and B can be chosen, and can either be good or bad. If the asset is good, it has a high outcome (e.g., +\$1.00) with probability $p > ½$ and a low outcome (e.g., \$0.00) with probability $(1 – p)$. If the asset is bad, it has the high outcome with probability (1 – p) and the low outcome with probability p.

Assume that n choices have been made, and the subject sampled n_A times from asset A and $n_B = n – n_A$ times from asset B. Let n_A^H denote the number of times the high outcome was drawn when asset A was chosen, and $n_A^L = n_A – n_A^H$ be the number of times the low outcome was drawn when asset A was chosen. Similarly, let n_B^H denote the number of times the high outcome was drawn when asset B was chosen, and $n_B^L = n_B – n_B^H$ be the number of times the low outcome was drawn when asset B was chosen. Then, given this information seen by the subject, the Bayesian posterior probability that asset A is the good one is given by: $Prob\{A = Good \mid n_A^H, n_A^L, n_B^H, n_B^L\} =$

$$\frac{1}{1+p^{n_A^L-n_A^H+n_B^H-n_B^L}*(1-p)^{n_A^H-n_A^L+n_B^L-n_B^H}}$$

The probability that asset B is the good one is given by:

$Prob\{B = Good \mid n_A^H, n_A^L, n_B^H, n_B^L\} = 1 – Prob\{\text{A} = Good \mid n_A^H, n_A^L, n_B^H, n_B^L\}$
