# Supplementary material for: Gain and Loss Learning Differentially Contribute to Life Financial Outcomes
Source: PLoS One. 2011 Sep 6;6(9):e24390. doi: 10.1371/journal.pone.0024390 (PMC3167846; doi:10.1371/journal.pone.0024390)
Supplement: Methods S2 — Calculation of risk aversion and loss aversion measures. (PDF) [file pone.0024390.s005.pdf]

Methods S2. Calculation of risk aversion and loss aversion measures.

Risk aversion was measured by subjects' switching point in the lottery choice questions below (i.e., the point at which they switched from choosing the sure outcome to choosing the gamble). Note that these items only included gambles in the gain domain. The higher is the value recorded (i.e., the later the subject switched from the safe to the risky choice), the more risk averse is the individual (i.e., the more concave is the utility function in the gain domain).

Which gambles would you be willing to take? (circle whichever alternative you prefer)

100% chance of winning $10	-vs-	50% chance of winning $0, 50% chance of winning $10
100% chance of winning $10	-vs-	50% chance of winning $0, 50% chance of winning $12
100% chance of winning $10	-vs-	50% chance of winning $0, 50% chance of winning $14
100% chance of winning $10	-vs-	50% chance of winning $0, 50% chance of winning $16
100% chance of winning $10	-vs-	50% chance of winning $0, 50% chance of winning $18
100% chance of winning $10	-vs-	50% chance of winning $0, 50% chance of winning $20
100% chance of winning $10	-vs-	50% chance of winning $0, 50% chance of winning $22
100% chance of winning $10	-vs-	50% chance of winning $0, 50% chance of winning $24
100% chance of winning $10	-vs-	50% chance of winning $0, 50% chance of winning $26
100% chance of winning $10	-vs-	50% chance of winning $0, 50% chance of winning $28
100% chance of winning $10	-vs-	50% chance of winning $0, 50% chance of winning $30
100% chance of winning $10	-vs-	50% chance of winning $0, 50% chance of winning $32

Loss aversion was measured by subjects' switching point in the lottery choice questions below (i.e., the point at which the subject switched from choosing the sure outcome to choosing the gamble). Note that these items included mixed gambles (i.e., in which both gains and losses were possible for small amounts around $0.00). Assuming a simple form of the utility function (e.g., U(x)=x if x>=0, and U(x)= lambda x if x<0, with lambda>0), as typically done in studies that seek to estimate loss aversion, the higher is the switching point, the higher is the subject's loss aversion (captured by parameter *lambda* in the utility function above).

Which gambles would you be willing to take? (circle whichever alternative you prefer)

100% chance of winning $0	-vs-	50% chance of losing $10, 50% chance of winning $10
100% chance of winning $0	-vs-	50% chance of losing $10, 50% chance of winning $12
100% chance of winning $0	-vs-	50% chance of losing $10, 50% chance of winning $14
100% chance of winning $0	-vs-	50% chance of losing $10, 50% chance of winning $16
100% chance of winning $0	-vs-	50% chance of losing $10, 50% chance of winning $18
100% chance of winning $0	-vs-	50% chance of losing $10, 50% chance of winning $20
100% chance of winning $0	-vs-	50% chance of losing $10, 50% chance of winning $22
100% chance of winning $0	-vs-	50% chance of losing $10, 50% chance of winning $24
100% chance of winning $0	-vs-	50% chance of losing $10, 50% chance of winning $26
100% chance of winning $0	-vs-	50% chance of losing $10, 50% chance of winning $28
100% chance of winning $0	-vs-	50% chance of losing $10, 50% chance of winning $30
100% chance of winning $0	-vs-	50% chance of losing $10, 50% chance of winning $32
